# Breaking Health Insurance Knowledge Barriers Through Games: Pilot Test of Health Care America

**DOI:** 10.2196/games.7818

**Published:** 2017-11-16

**Authors:** Sara Champlin, Juli James

**Affiliations:** ^1^ Mayborn School of Journalism The University of North Texas Denton, TX United States

**Keywords:** health insurance, games, experimental, young adult, information literacy

## Abstract

**Background:**

Having health insurance is associated with a number of beneficial health outcomes. However, previous research suggests that patients tend to avoid health insurance information and often misunderstand or lack knowledge about many health insurance terms. Health insurance knowledge is particularly low among young adults.

**Objective:**

The purpose of this study was to design and test an interactive newsgame (newsgames are games that apply journalistic principles in their creation, for example, gathering stories to immerse the player in narratives) about health insurance. This game included entry-level information through scenarios and was designed through the collation of national news stories, local personal accounts, and health insurance company information.

**Methods:**

A total of 72 (N=72) participants completed in-person, individual gaming sessions. Participants completed a survey before and after game play.

**Results:**

Participants indicated a greater self-reported understanding of how to use health insurance from pre- (mean=3.38, SD=0.98) to postgame play (mean=3.76, SD=0.76); *t*_71_=−3.56, *P*=.001. For all health insurance terms, participants self-reported a greater understanding following game play. Finally, participants provided a greater number of correct definitions for terms after playing the game, (mean=3.91, SD=2.15) than they did before game play (mean=2.59, SD=1.68); *t*_31_=−3.61, *P*=.001. Significant differences from pre- to postgame play differed by health insurance term.

**Conclusions:**

A game is a practical solution to a difficult health issue—the game can be played anywhere, including on a mobile device, is interactive and will thus engage an apathetic audience, and is cost-efficient in its execution.

## Introduction

Serious games are designed to cultivate skills for a specific topic and do so by activating existing schema in the mind of a player to ultimately produce “new knowledge” and experiences [1]. In contrast to reading a pamphlet or searching online, players engage with serious games, given their inherent fulfillment of a need to have fun or be satisfied, as suggested by self-determination theory [2]. As such, serious games are increasingly implemented to train or immerse players in novel experiences so that they may encounter and test structures and concepts in an enjoyable way. In turn, players often exhibit increased conceptualization of specific challenges, even for stigmatized health issues such as sexual risk [3]. In addition to building skill sets, games can increase self-efficacy and result in behavior change [4]. Given these benefits, serious games are a growing, effective medium for current and future generations of young adult audiences [5].

There exists a variety of health-oriented serious games. In some cases, health-focused games allow players to explore risky or challenging situations without having to experience the direct effects they might encounter in the real world (eg, dying or harming someone, experiencing a negative health outcome, mismanaging a decision) [6]. This includes training and allowing players to practice specific skills such as attention and reaction time while driving [7] or learning and experiencing how to prepare surgical instruments [8]. Other games emphasize education and promoting an increased understanding of action-oriented knowledge. For example, after playing a game titled Mommio, mothers indicated that they acquired knowledge by experiencing interactions between a mother and a child who does not enjoy eating vegetables [9]. Other games depict topic-specific knowledge increases, such as increased information about nutrition as a result of playing a diet- and exercise-oriented game titled SpaPlay [10].

A key benefit of serious games for health, specifically, is to stimulate or motivate patients for health efforts that may be perceived as unpleasant or uninteresting and thus avoided [6]. Games can also help players understand specific positions or roles in one’s life, akin to imagining what it would be like to try on a specific identity [6]. Games of every kind allow players to take on roles outside of their everyday lives, which can have positive impact [11,12].

According to the Entertainment Software Association, 56% of Americans today play video games, and nearly half (48%) of frequent gamers are considered mobile and/or social gamers [5]. Moreover, 77% of college-aged men and 57% of college-aged women report playing video games [13]. As such, digital and Web-based games are a reasonable and strategic platform to engage with these audiences. Games present an additional opportunity to connect audiences with news and with health. The overlapping, growing trends in gaming and media speak to the opportunity for using a mobile-ready Web-based game to engage new demographics in meaningful health information in innovative ways, reach existing audiences with alternative, interactive journalism, and speak to a new generation of media users on the devices and in the media with which they are already interacting.

In this study, we developed a serious game that draws from principles of journalism, educational and video game design, and learning and literacy, in conceptualizing an online game experience [14,15]. Given that young adults may currently avoid or misunderstand health insurance information, a game is a practical solution to this difficult health issue. A Web-based health game may be particularly appropriate for young adults, almost all of whom use the Internet. The game can be played anywhere, including on a mobile device, is interactive and will thus engage an apathetic audience, and is cost-efficient in its execution.

### Health Insurance Literacy

Having health insurance has been linked to a number of advantageous health outcomes, including increased access to health monitoring, screening, information, and beneficial health decisions [16]. As a result, those who do not have health insurance are at a greater risk for serious and exacerbated health consequences related to cancer, cardiovascular issues, hypertension, diabetes, kidney disease, human immunodeficiency virus infection, and injury [16].

Understanding, selecting, and using a health insurance plan, however, is a complex process and requires a plethora of diverse literacy and numeracy skill sets [17]. Politi and colleagues argue that health insurance plan comprehension and decision making are “essential” for the consumer [18]. Thus, determining ways to discuss and facilitate health insurance information with the public is an important avenue for health-based research. Simultaneously, patients are increasingly encouraged to take ownership of their own health information and participate in their own health care [19-21]. As a result, patients are given a growing amount of health insurance information and details to interpret and consider [22].

Although the initiation of the Affordable Care Act increased the number of Americans who hold health insurance policies [23], it is not yet known whether health insurance consumers indeed have knowledge and confidence in how to use their benefits [24]. Previous research suggests that insured adults lack an understanding of terminology and how to use this important entity. Many states have implemented the use of health insurance counselors to aid consumers in their understanding and selection of a health insurance plan. Interviews with counselors point to critical health insurance literacy concerns; patients tend to avoid reading information about specific plans and, as a result, misunderstand how much coverage they have or how much they owe as the result of a medical procedure (eg, if the patient pays a premium, the patient might think he or she does not owe any additional medical costs) [25]. Counselors also note that patients rely on word-of-mouth information about plans; often misunderstand terms such as “co-pay,” “deductible,” and “co-insurance”; and lack knowledge about provider restrictions such as being in network/out of network [25].

Historically, young adults have the lowest rates of health insurance plan enrollment among all nonelderly adult age groups [26,27]. Young adults remain the age demographic with the fewest insured individuals; 14.4% of young adults aged between 18 and 24 years did not have health insurance in 2015 and 17.9% of those aged between 25 and 34 years [28]. Limited research has specifically tackled strategies for best communicating health insurance information to this vulnerable population. It is worrisome that young adults may not be equipped with the knowledge or skills to appropriately select and enroll in a plan. Furthermore, young adults with plans may struggle when navigating the health care system and amass high health care bills.

Health insurance literacy is particularly low among young adults, including those who are highly educated. In one study, none of the participants felt “good” or “very good” about their understanding of a list of health insurance terms and at times transposed definitions or had questions about the information [29]. The need for increased understanding about health insurance among young people is a long-standing health challenge. Robertson and Middleman called for greater health insurance education for young people based on their findings that almost half of the adolescents in their study were unaware of “how their medical bills [were] paid” [30]. Young adults should be equipped with the information necessary for them to make informed decisions when it comes to getting and using health insurance.

### Newsgames

For this project, we created a Web-based newsgame, “Healthcare America,” to explore the possibilities for what games can offer the problem of engaging and informing young adults in concepts connected to health insurance literacy. Bogost and colleagues define “newsgames” as games that apply journalistic principles in their creation [14]. For example, a newsgame might implement stories from various first-person sources or illustrate the complexities of a real-world issue.

Specifically, newsgames infuse the concept of real-world *narrative* into game play. Narrative communication and storytelling are promising methods to increase engagement of an audience with content, yet are not frequently utilized in health-related research and interventions [31]. Thompson and Kreuter note that “vivid, engaging writing can help audiences identify with storytellers and understand health messages” [32]. A growing interest in research is the narrative work written by medical professionals, which can promote improved on-the-job skills when read and considered by other professionals working in this industry (ie, learning from someone else through their stories) [33]. It was the hope of this study that playing a game with client-based narratives generated from news and real-world encounters and interviews would contribute to increased understanding of health insurance information.

Newsgames are an emerging digital practice for today’s journalists, although games for journalism are not new. Historically, crossword puzzles and news quizzes have been found in newspapers and on broadcast radio as tools to engage audiences in interacting with news facts and information in engaging and fulfilling ways. As video games have evolved and the benefits of game design have grown out of commercial markets into serious and educational spaces, journalism has also begun taking the best practices of game design for nuanced storytelling, immersive learning and informing strategies, and engaging audiences in complex systems through play [34].

We selected a newsgame approach for Healthcare America to explore a mobile game execution for health journalism and to experiment with and contribute to the design models for engaging journalism. Games at the intersection of journalism and health provide new, strategic opportunities to create playful experiences around complex issues and offer real-world learning created from real data designed into hypothetical interactive narratives. These serious games provide a platform for audience engagement in ways that health communication and health journalism have only just begun to explore (conventional tools in health media communication include pamphlets and flyers, public service announcements, and radio ads) [11].

The purpose of this study was to develop an interactive-narrative newsgame that presents entry-level health insurance information and scenarios to young adults. To our knowledge, this study is the first to design and test an interactive game that encourages young adults’ interaction with health insurance concepts.

We hypothesized the following statements based on the notion that young adults often lack an understanding of many health insurance terms [29] and exposure to an interactive game about health insurance will improve this knowledge; given this, after game play:

*Hypothesis 1*: Participants will exhibit a greater self-reported, general understanding of what health insurance is and how to use health insurance.

*Hypothesis 2*: Participants will exhibit increased self-reported understanding of critical health insurance terminology.

In addition to examining whether participants perceived themselves to have learned about health insurance terminology from the game, we sought to measure whether participants increased in their objective knowledge of health insurance terminology after game play.

*Hypothesis 3*: Participants will be able to correctly define a greater number of health insurance terms following game play.

Findings from this study will have important implications for many groups. Equipping young adults with a greater understanding and confidence in what health insurance is and how it works can lead to better overall health outcomes. Additionally, this study offers practical solutions for health practitioners, educators, and counselors who design programs to improve health literacy and access to health information. In the remainder of this paper, the development of a health insurance game created for young adults will be described and its effects tested. Implications for health practitioners, including health insurance counselors and other financial advisors, will be discussed.

## Methods

### Game Development Tool

We used the Playable Media Story Builder (Phoenix, Arizona) platform to develop the game, Healthcare America. The Story Builder is a visual engine that allows publishing of hosted, cross-platform responsive, interactive narrative games. The engine, designed to empower journalists to prototype narrative games, was funded by the Knight Foundation Knight Prototype Fund. The Playable Media team designed and developed the tool in collaboration with journalists and journalism students at Arizona State University through the News-Play Project, a partnership between Arizona State University’s New Media Entrepreneurship & Innovation Lab and Center for Games & Impact Innovation Lab.

### Game Design and Development

**Figure 1:**
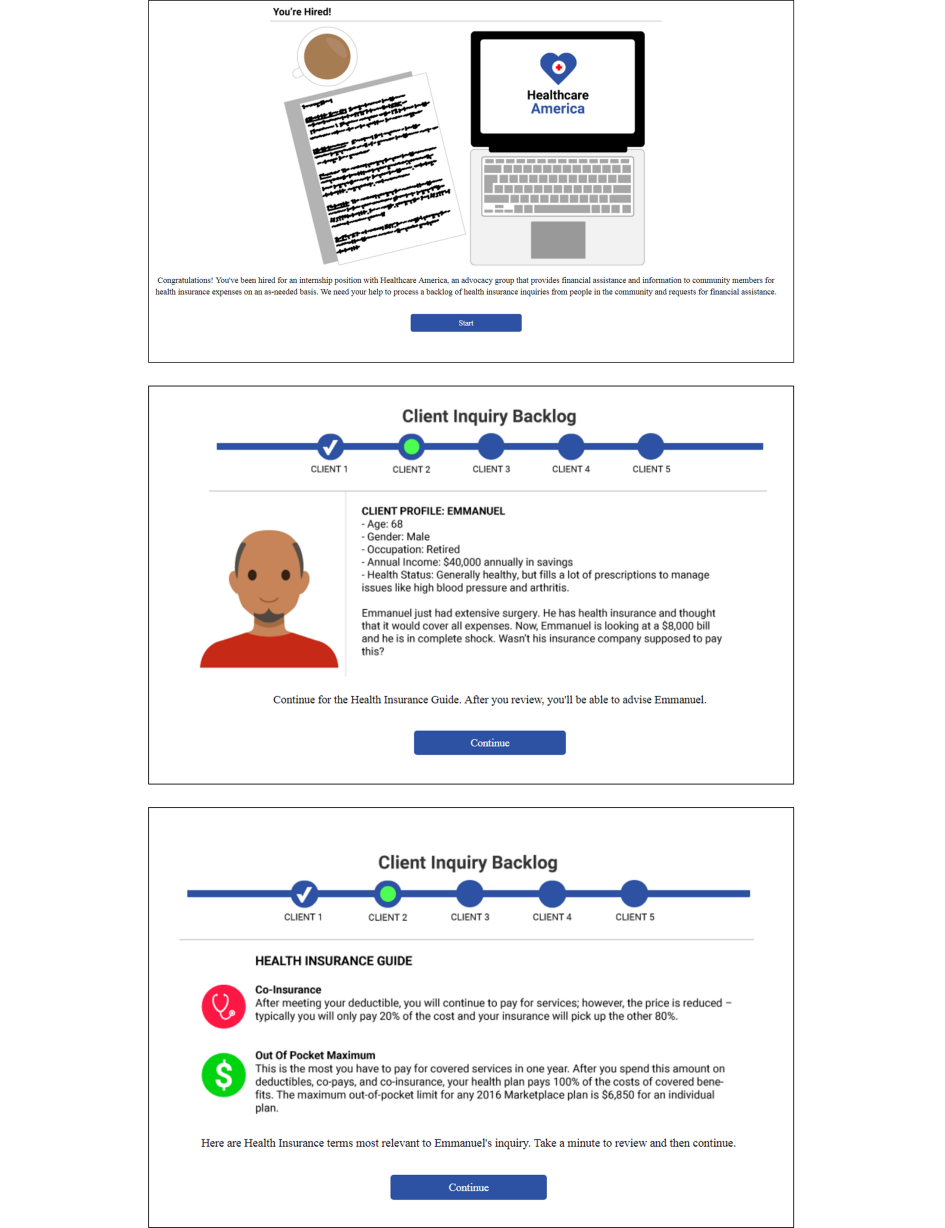
Examples of the user interface and game play of the health insurance game.

Healthcare America positions the player as working for a health information advocacy organization with a series of client cases. The game interface includes simple graphics, narrative text, player choices, and two meters measuring community wellness and health care assistance funds (see Figure 1).

Using a newsgame design approach to develop game content, we gathered stories about personal struggles with health insurance from people in our community, undergraduate students, and national news stories. We collaborated with a news writing and reporting class to further investigate through personal narratives the ways in which people struggle with health insurance in the real world. Additionally, we pulled fact-based information from health insurance websites, the HealthCare.gov website, and our university’s health insurance Web page. We collaborated with stakeholders and knowledge experts at our university about how they typically administer information and guidance about health insurance to students.

As a result of this exploration, five scenarios or “client cases” were developed for the game based on real-world stories and information gathered from stakeholders and health insurance consumers. Characters included in the game were developed to consciously include a variety of different race/ethnicities, sex (white female, African American male, Hispanic female, white male, Asian female), age, and occupation.

Following these steps, we adapted the list of health insurance terms used by Wong and colleagues [29] to include the following: “the Affordable Care Act,” “deductible,” “monthly premium,” “referral,” “in-network provider,” “co-payment,” “out-of-pocket maximum,” “coinsurance,” and “health maintenance organization (HMO) and preferred provider organization (PPO).” Within the game, each term and a corresponding definition was provided to the participant for their review. See Figure 1 for a screenshot of two definitions, which were both relevant for a case in which a consumer in the game had recently encountered unexpected health insurance charges.

The game opened with a brief introduction and explanation of the game on screen. Game instructions, including the meaning of the game meters (Community Wellness, that is, how healthy the participant or player made the community, and Healthcare Assistance Fund, that is, the amount of money spent to assist the client), were then provided. With these meters in mind, the central goal of the game was to be a “successful intern” by assisting clients with troubling health insurance cases—aiming to maximize Community Wellness without spending all of the organization’s Healthcare Assistance Fund.

Participants read and experienced each of the aforementioned cases; key details about what happened and what went wrong for the client were presented. Then, health insurance terms were provided. Participants then selected one of three options for how to advise each client, which took into account how much each option would contribute to the overall Community Wellness (Did the participant help improve the health of the community through his or her advising decision?) and how much money would be spent from their Healthcare Assistance Fund, which was capped at US $5000.

To summarize and provide a specific example, the prototype narrative design of Healthcare America blends real-world experiences of stories from using, or struggling to use, health insurance. In the game, these stories are translated to “client cases.” The player is given a role within the narrative and the goal of “solving” or assisting a series of client cases while working as an intern (with the promise of being promoted to a full-time position for a job done well).

The player reads a client case file (eg, Vanessa who is a 42-year-old female, a project manager and mother of 2, annual income US $35,000, who regularly sees a therapist to manage her mental health and well-being. She fills a prescription for antidepressants on a monthly basis. “Vanessa sent us [Healthcare America] an email because she’s confused about what just happened at the pharmacy. After being diagnosed with depression, Vanessa has a regular prescription for antidepressants. Today the cost of her medication was much higher than usual.”) and is then provided with information about specific health insurance term (in the case of “Vanessa,” it is information about co-payments). The player is then able to advise the client using several options, each of which is linked to a corresponding increase or decrease in the Community Wellness meter, the Healthcare Assistance Fund, or both. The player advises the client presented and then learns the outcome of the corresponding meters.

**Figure 2:**
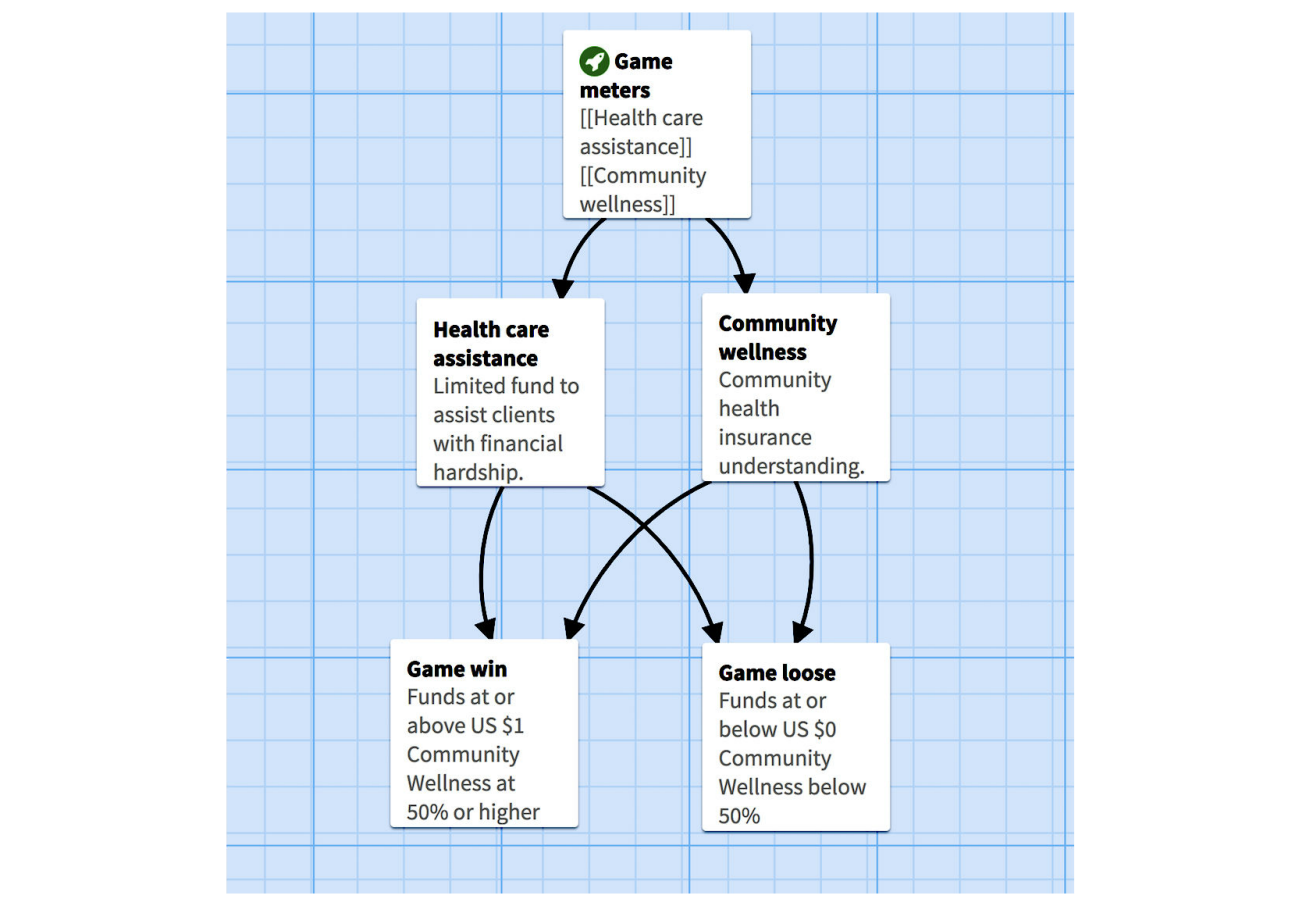
Design of game meters: (1) Healthcare assistance fund and (2) community wellness.

**Figure 3:**
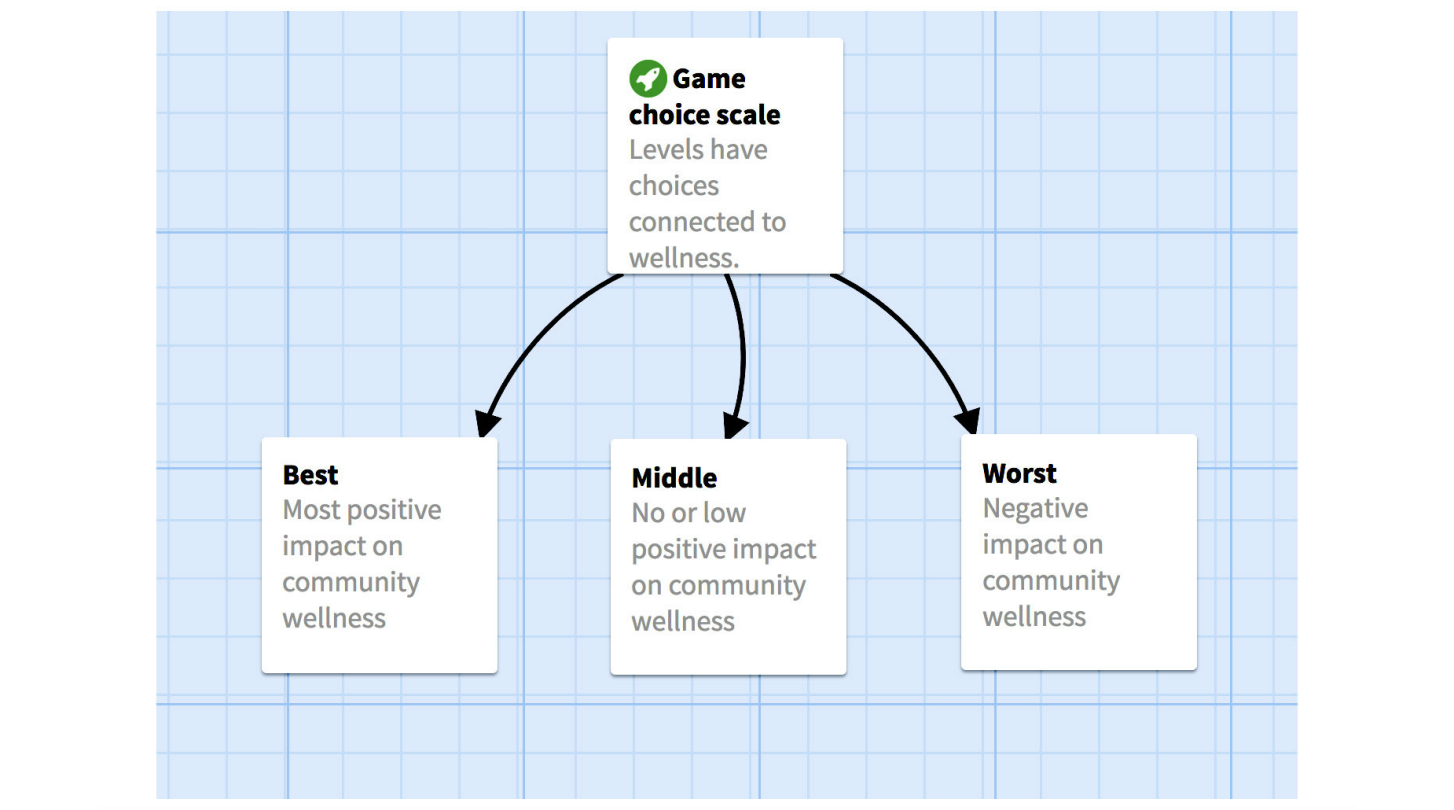
An example of the community wellness meter options.

There are obstacles to overcome (the main conflict is to balance solutions to clients’ health care and health insurance problems) and rewards for overall Community Wellness and the Healthcare America’s Healthcare Assistance Fund. The game ends if the player depletes the Community’s Wellness or Healthcare Assistance Funds as a result of his or her decision to assist a client (a loss) or if the player successfully assists all 5 clients (a win); see Figures 2, 3 and Multimedia Appendix 1.

We kept in mind the *body of information* as is needed both for a narrative driven game and for narrative-driven journalism, and *situated* the information a player would need to use [35,36]. As game designers, we designed each level of Healthcare America based on a health insurance problem and distributed the essential information the player would need across the game to overcome obstacles and work toward the goal at the start of each level to gauge how much health insurance term information the player would need and could use—just enough information and just in time [36].

**Table 1:** Demographic information of sample (N=72).

Characteristic		Sample
**Sex, n (%)**		
	Male	29 (40)
	Female	43 (60)
**Race/Ethnicity, n (%)**		
	White	27 (38)
	Black, African American	18 (25)
	Hispanic, Latino/a	17 (24)
	Asian	5 (7)
	Mixed	4 (6)
	Other	1 (1)
**First-generation college, n (%)**		
	Yes	19 (26)
**General health, n (%)**		
	Excellent	8 (11)
	Very good	30 (42)
	Good	27 (38)
	Fair	5 (7)
	Poor	1 (1)
	Don’t know	0 (0)
**Academic year, n (%)**		
	Freshman	19 (26)
	Sophomore	12 (17)
	Junior	26 (36)
	Senior	10 (14)
	Super senior (+4 years)	3 (4)
	Other	1 (1)
**Non-English household, n (%)**		
	Yes	17 (24)
Without health insurance, n (%)		17 (24)
Age in years, mean (SD)		21.15 (3.49)

### Participants

A 2-tailed power analysis calculated for paired data was performed with G-Power to calculate an estimated sample size of 67 participants (effect size=0.35, with 80% power, and alpha=.05) [37]. In total, 75 (N=75) students were included in this study. Due to a computer error, three of these participants were unable to play the game and were thus removed from the dataset, resulting in 72 (N=72) valid participants. The demographic information of the sample is included in Table 1.

### Procedures

A call for participants was administered via email to classrooms, or a Web-based classroom boards through journalism and communications courses on campus. Participants indicated their interest by contacting the research team via email and scheduling an appointment. Each participant completed an individual, in-person data collection session. All study procedures were approved by the relevant institutional review board. Each session was scheduled for 1 hour and included an introductory phase in which participants read and signed a consent form, the completion of a pregame survey, game play on a computer, and a postgame survey.

### Measures

Participants completed pre- and postgame surveys to determine their level of health insurance understanding before and after playing the health insurance game. In both surveys, participants were asked to indicate their understanding of “what health insurance is” and “how to use health insurance,” as well as rate their understanding of 9 health insurance terms using a 5-point scale (very good to very bad) [29]. Additionally, to measure objective understanding of a given health insurance term, participants were asked to write a definition for each of the 9 health insurance terms. These items were adapted from previous research [29]. Similar to Wong and colleagues, a team of 4 investigators determined whether each health insurance term was defined correctly or incorrectly based on the content provided in the game and the definitions provided by health insurance companies and Healthcare.gov. Participants also completed demographic items, including sex, race/ethnicity, age, whether they were a first-generation college student (the first in their family to attend college), self-reported rating of their general health, year in school, whether they currently have health insurance, and whether they grew up in a household that primarily spoke a language other than English. In cases where multiple numerical responses were selected (eg, both 3 and 4 to indicate understanding of a health insurance term), the average of the 2 numbers (eg, 3.5) was used.

### Analyses

Paired sample *t* tests were used to determine whether participants’ self-reported understanding of what health insurance is, how to use health insurance, and the 9 health insurance terms were different between pre- and postgame play. A paired sample *t* test was also used to determine whether the total number of correctly defined health insurance terms differed from pre- to postgame play. A Cochran’s Q test was used to examine differences in correctly defined health insurance terminology (paired nominal data) between pre- and postgame play.

## Results

The purpose of this study was to determine whether a health insurance game is a viable tool to communicate important information about health insurance terminology and how health insurance is used. The first hypothesis predicted that, following game play, participants would exhibit a greater self-reported, general understanding of what health insurance is and how it is used. Indeed, participants indicated a greater self-reported understanding of how to use health insurance from pregame play (mean=3.38, SD=0.98) to postgame play (mean=3.76, SD=0.76); *t*_71_=−3.56, *P*=.001. There was no significant difference between pre- and postgame play for participants’ self-reported understanding of what health insurance is. Given this, hypothesis one was partially supported.

Hypothesis 2 surmised that participants will exhibit increased self-reported understanding of critical health insurance terminology. For all health insurance terms, participants felt they had a better understanding of the term following the game; see Table 2 for complete results.

Finally, in addition to examining whether participants felt that their understanding of a given health insurance term increased after playing a health insurance game, we wanted to determine whether participants objectively define more health insurance terms correctly following game play. The proportion of participants who correctly defined a given health insurance term was significantly different for the following terms: “monthly premium,” “referral,” “in-network provider,” “deductible,” and “HMO/PPO”; see Table 3 for complete results.

Among participants who provided a definition for every health insurance term for both pre- and postgame play (N=32), participants correctly defined more health insurance terms following game play (mean=3.91, SD=2.15) than they did before game play (mean=2.59, SD=1.68); *t*_31_=−3.61, *P*=.001. However, the number of correct definitions provided following game play did not correlate with participants’ overall self-reported understanding of all terms following game play (self-reported scores for all terms were summed together), *r*=.18, N=42, *P*>.05.

**Table 2:** Self-reported understanding of health insurance terms, pre- and postgame play.

Health insurance term	Pregame self-reported understanding, mean (SD)	Postgame self-reported understanding, mean (SD)	Paired *t* statistic (degrees of freedom)^a^	*P* value
Affordable Care Act	2.75 (1.12)	3.73 (0.81)	−6.92 (70)	<.001
Premium	2.70 (1.20)	3.87 (0.81)	−8.89 (70)	<.001
Referral	3.11 (1.37)	4.13 (0.81)	−6.25 (70)	<.001
In-network provider	2.35 (1.37)	3.71 (0.97)	−8.58 (71)	<.001
Co-pay	3.24 (1.32)	3.72 (1.03)	−3.10 (68)	.003
Deductible	2.97 (1.17)	3.77 (0.80)	−5.06 (70)	<.001
Out-of-pocket maximum	2.23 (1.18)	3.39 (1.09)	−7.62 (69)	<.001
Coinsurance	1.91 (1.00)	2.39 (1.03)	−3.88 (68)	<.001
Health maintenance organization and preferred provider organization	1.97 (1.04)	3.26 (1.09)	−9.10 (68)	<.001

^a^Degrees of freedom differ across terms given that participants had missing data on either pre- or postgame items at different rates for each term.

**Table 3:** Objective understanding of health insurance terms, pre- and postgame play.

Health insurance term	Correct pregame, n (%)	Correct postgame, n (%)	Cochran’s Q test statistic (degrees of freedom)	Asymptotic *P* value
Affordable Care Act	15 (25)	17 (29)	0.33 (1)	.56
Monthly premium	27 (46)	39 (66)	5.54 (1)	.02
Referral	19 (31)	43 (70)	18.00 (1)	<.001
In-network provider	19 (40)	34 (71)	13.24 (1)	<.001
Co-payment	25 (41)	23 (38)	0.15 (1)	.70
Deductible	7 (12)	23 (40)	12.80 (1)	<.001
Out-of-pocket maximum	13 (25)	11 (22)	0.25 (1)	.62
Co-insurance	0 (0)	3 (7)	3.00 (1)	.08
Health maintenance organization and preferred provider organization	2 (4)	18 (39)	14.22 (1)	<.001

## Discussion

### Principal Findings

Findings from this study suggest that the implementation of a newsgame can facilitate health insurance knowledge among young adults, a population vulnerable to the negative impact of not having or understanding health insurance [27-29]. In this study, a Web-based newsgame about health insurance was developed by exploring health insurance challenges experienced by everyday consumers and re-creating these scenarios in the context of an interactive game.

Young adults, even those who are well-educated, struggle with understanding health insurance and health insurance terminology [29]. On the basis of the findings from this study, a game appears to be a viable option for presenting this audience with information about this challenging topic. This aligns with the existing research on the beneficial impact of narrative on health, decisions, and skills [31-33]. Following game play, participants self-reported a significantly greater understanding for all terms. It is encouraging that participants felt that they understood more of this difficult content, as previous research notes that evading health insurance information is common [25]. It may be that the medium, a gaming platform, combined with a narrative newsgame approach creates a context in which emerging adults feel comfortable, in contrast to pamphlets or static websites. Upon examining objective knowledge assessment through correctly/incorrectly defined health insurance terms, however, it is important to note that participants made significant improvements for some, but not all, health insurance terms.

In this study, significantly more participants provided correct definitions for the terms “monthly premium,” “referral,” “in-network provider,” “deductible,” and “HMO/PPO” following game play. The largest increase was seen for the term “referral,” where the frequency of correct definitions increased by 39%. It is possible that this is a term for which many students were already familiar with but needed to be reminded of the correct definition. Indeed, many of the top reasons why college students visit a health care provider are for health concerns that require a referral or prescription including respiratory infections, sexually transmitted diseases, birth control, and annual exams (mainly women’s health such as pap smears) [38], which could explain why referral garnered the largest increase from pre- to postgame play.

In contrast, HMO and PPO are likely terms with which college students are not familiar with [29], yet a significant improvement was observed in this study. Indeed, before game play, only 2 participants in this study correctly defined this concept. A total of 18 participants (39%, 18/46) demonstrated an objective understanding of “HMO” and “PPO” after playing the game. In addition to this being the last term presented (and thus closest to the administration of the post-game survey), this effect could be due, in part, to the relatability of the client profile to the student participants. The client featured in the game regarding the “HMO/PPO” term admitted to being “new to having health insurance,” was relatively young (33 years old) and worked as a communications professional at a start-up company. Although the focus of this study was not on the relationship or relatability of the client/profile with the participant, perceptions of homophily would be a fruitful focus for future research when it comes to increasing engagement with health insurance information.

Although significant growth was not observed in the number of participants correctly defining the term “coinsurance” between pre and postgame play, it is worth noting that no participant provided a correct definition before playing the game. Following the game, 3 participants provided a correct definition. On the basis of the findings of this study, of the terms provided in the Healthcare America game, “coinsurance” requires the greatest exploration in future initiatives. This aligns with previous research, which suggests that patients often focus on the concept of paying premium every month but neglect other cost-sharing terms, including “coinsurance” [25]. “Coinsurance” may be commonly complicated with “co-payment” and perhaps “cost-sharing” and thus should be teased apart further in future initiatives. In this study, all terms were given equal attention (ie, presented an equal number of times, one opportunity to make a decision related to the term rather than repeated practice), yet findings suggest that future initiatives attribute greater explanation and additional examples for some terms.

Akin to Wong and colleagues, the correlation between the number of correctly defined health insurance terms and participants’ self-reported understanding of all terms was nonsignificant [29]. One explanation for this discrepancy is that, although participants may feel that they understand health insurance content (self-report knowledge), *providing a definition* (objective knowledge) is notably more challenging. Although writing a correct definition served as a valuable method for assessing participant knowledge for the purpose of a research study and has been used in previous research [29], this is not how health insurance knowledge would be captured in everyday activities. Rather, individuals must make decisions using the given information, including completing calculations. It could be that participants would have been more successful in demonstrating their knowledge through applied scenarios and questions, comparable with what they did within the game. This is just one of many avenues for future research initiatives.

Additionally, in this study, participants indicated an increased self-reported understanding of how to use health insurance but not what health insurance is. In designing the game for this study, it became clear that there are a number of avenues to be tackled through games when it comes to health insurance. Future research should explore the needs of those who do not yet have health insurance and how gaming, immersion, and decision making can contribute to enrollment assistance. In conjunction with this initiative, those who currently have health insurance, yet struggle with using the health care system and insurance companies, would benefit from a game similar Healthcare America. Future gaming efforts could incorporate both suggestions into one game, having participants reach or unlock new levels as they successfully move from getting enrolled in health insurance initially, to making challenging decisions in a health care setting.

It should be noted that Healthcare America is just one example of how games can be used successfully to promote health knowledge and outcomes. There are a growing number of examples in which games and game projects are designed to engage audiences in health and wellness issues. Indeed, UnitedHealthcare offers a Health Insurance Matchmaking Game in which the player determines which type of health insurance is “right” for them based on a series of actions performed in the game [39]. Future iterations of this study could compare the effectiveness of Healthcare America with other games and strategies for increased interaction with and understanding of health insurance. In contrast, at the specific intersection of health games and health journalism, in which stories and scenarios from the real world are directly incorporated into the design of the game, there are only limited projects despite the possibilities for increased awareness, engagement, and education. *Seven Ways to Defy Death* from the Washington Post [40] does provide one example, as well as Propublica’s experimental game, *Heartsaver* [41]. Journalists cite time, design and technical resources, and budget as pain points for developing games to engage audiences in game-infused storytelling. Free engines such as the Story Builder used for this project offer journalists the ability to create mobile games quickly and experiment with audience engagement in this space.

The use of a Web-based platform, such as that used in this study, has far-reaching implications. This is especially clear in context of growing mobile “smartphones,” which are capable of accessing the Internet. Nearly all (92%) of Americans aged 18 to 29 own a smartphone [42]. As such, dissemination of our health insurance newsgame could be simple and extensive. The game could be texted to a phone number or the link sent through email, and shared via social platforms such as Facebook, Twitter, Instagram, or Snapchat, without the need for in-person game administration assistance. One avenue for future research would be the implementation of this game in college campus health and wellness center waiting rooms, where students may actively desire something to fill their time while waiting. Using a Web-based, mobile-ready game also provides the potential for reaching populations underserved in health insurance understanding; younger adult audiences may not get their news and information from traditional outlets, but this is a large population of the people who are online, on mobile technologies, and are playing games. There are also possibilities for game-infused programs for health insurance literacy that begin with the Healthcare America mobile game as part of a suite of health insurance literacy games that deploy automatically to participants at particular times during their university experience and incorporate participant follow-up surveys along the way possibly between 3 and 6 months, at 1 year, and at university exit/graduation. This strategy addresses the issue of building real literacy in this complex system, realizing it is not a problem that can be solved with one game, one time.

In addition to these explicit advantages with a young, tech-savvy population, the majority of adults with potentially low health literacy indeed have smartphones including those who did not graduate from high school (54% have smartphones), make less than US $30,000 (64%), or are of minority race (72% of black adults and 75% of Hispanic adults). Future research initiatives should explore ways in which a newsgame would be a feasible method for connecting patients with low health literacy, general literacy, or experience difficulty with technology. One adjustment that could be made to Healthcare America is to offer audio cues and narration to facilitate engagement among these audiences. Additionally, game content could be further tailored to specific populations and include culturally relevant details, as well as specific health insurance scenarios encountered by patients who struggle with these skill sets.

### Limitations

This study presents important findings that can contribute to future intervention initiatives regarding health insurance, health, and newsgames, yet it is not without limitations. First, the study was limited by sample size. The purpose of this study was to explore an initial iteration of a newsgame in this area, and our study indeed exceeds the sample size included in other, comparable studies [18,29]. In Healthcare America, players are provided with information about and definitions of health insurance terms. Information is not sufficient to change behavior [43,44]. However, in conjunction with an interactive game in which young adults were asked to note, evaluate, and assist clients with health insurance issues, it is the hope that players experience and learn about health insurance through modeling and applying information they acquire. An additional limitation was the confines of the lab setting as well as participant self-selection (and convenience sampling) in choosing to play the game and participate in the study. It may be that participants felt they needed to respond to questions or act a specific way when playing the game. Participants could have felt pressured to take on the role of an “intern” for a health care position. With this in mind, within the game, the player is provided with the “pages from the intern manual” (the health insurance terminology explanations and examples) and thus must act and provide suggestions based on the information they have—a learning and growing process akin to working at an internship. In this study, we implemented previously used survey items [29]; however, other types of measures, including multiple-choice questions or selecting a “correct” answer from a list could have produced different results. Moreover, the findings rely on a single group pre- and postgame play design and did not measure subsequent behavior change or retained knowledge long term. In an effort to minimize these limitations in future studies, we are currently collaborating with the on-campus health and wellness center to make the Web-based game available on its website and thus extend access and data collection.

### Conclusions

In this study, young adult participants indicated an increased level of self-reported and objective knowledge after playing an interactive, narrative-based newsgame about health insurance. This population is particularly susceptible to being at risk for not having or understanding health insurance [27,29]; yet, based on the findings of this study, a game such as Healthcare America is an appropriate initial step to decreasing these health-related challenges.

Results from this study suggest that repeated play or multiple exposures to some concepts such as “coinsurance” may be needed to increase understanding of this more difficult term. In this study where only one example was implemented, few young adults increased in their understanding and confidence with this term. In contrast, terms that young adults encounter more often (and potentially have an increased immediate need for) such as “referral” need fewer examples or iterations to achieve understanding. Moreover, Healthcare America implemented client narratives based on real-world news stories, user experiences, and real-time information about health insurance to facilitate understanding. The strategy of asking young adults to help or assist others in need or with questions can be a fulfilling experience for the player and a beneficial game strategy.

Although the games for health field have grown considerably in recent years, this health insurance newsgame is the first of its kind. The design and pilot test phase shows promising results for game design strategy, health insurance content, and platform iterations, moving forward.

## Abbreviations

HMO: health maintenance organization
PPO: preferred provider organization

## Appendix

Multimedia Appendix 1 Full game design and possible game flow paths.
