# Supplementary figures and images for: Breaking Health Insurance Knowledge Barriers Through Games: Pilot Test of Health Care America
Source: JMIR Serious Games. 2017 Nov 16;5(4):e22. doi: 10.2196/games.7818 (PMC5709658; doi:10.2196/games.7818)

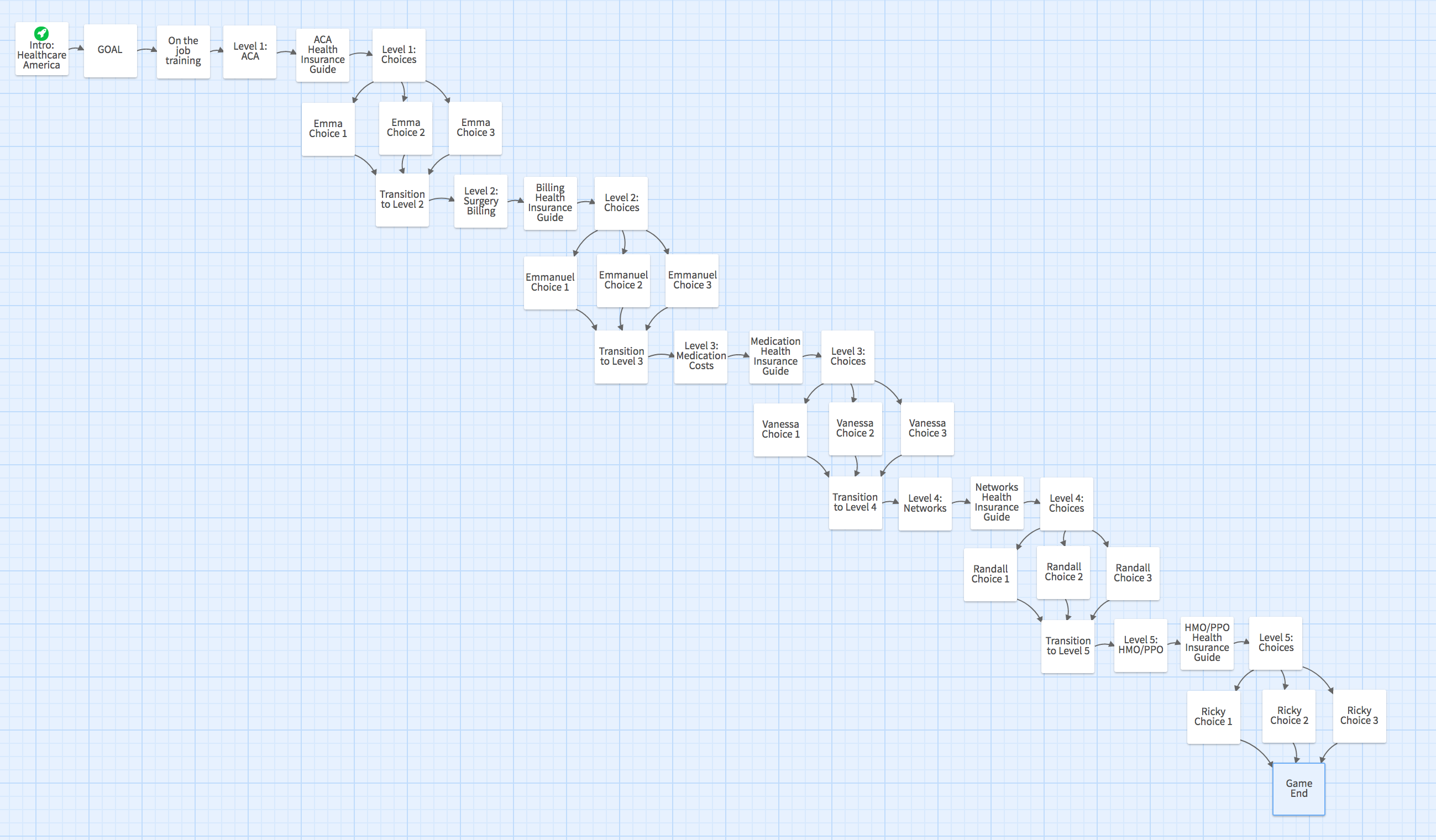

Supplement: Multimedia Appendix 1 [file games_v5i4e22_app1.png]
